# Intradiaphragmatic Bronchogenic Cysts: Case Report and Systematic Review

**DOI:** 10.1186/s13019-016-0444-9

**Published:** 2016-05-05

**Authors:** Ronnie Mubang, John Joseph Brady, Melissa Mao, William Burfeind, Matthew Puc

**Affiliations:** Department of General Surgery, St Luke’s University Health Network, Bethlehem, PA USA; Department of General Surgery, Philadelphia College of Osteopathic Medicine, Philadelphia, PA USA; Department of Thoracic Surgery, St Luke’s University Health Network, Bethlehem, PA USA

**Keywords:** Diaphragm, Bronchogenic cysts, Bronchogenic, Intradiaphragmatic

## Abstract

Bronchogenic cysts (BC) are congenital abnormalities that occur most commonly within the mediastinum, and rarely occur within the diaphragm. We present the 21st case of an intradiaphragmatic bronchogenic cyst in the English literature, and review all previous published cases. Analysis includes presenting clinical symptoms, relevant radiologic studies, surgical approaches to resection, and management of the diaphragm, among other relevant data. These lesions should remain on the differential diagnosis in cases of unusual masses in the region of the diaphragm.

## Background

Bronchogenic cysts (BC) are congenital abnormalities that arise from the ventral foregut and occur most commonly within the mediastinum. Bronchogenic cysts rarely occur within the diaphragm. Up to June 2015, there were twenty cases of intradiaphragmatic bronchogenic cysts reported in the English literature. We present a case of bronchogenic cyst located within the diaphragm in an adult male patient. This lesion was removed via a left thoracotomy approach and diagnosis was confirmed with surgical pathology.

### Case

**Fig. 1:**
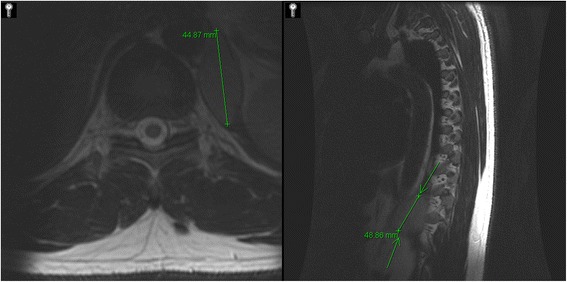
Axial and sagittal slices of MRI thoracic spine without contrast denoting a 5 cm × 4.5 cm ovoid hyperintense soft tissue structure medially at left lung base possible a bronchogenic cyst

**Fig. 2:**
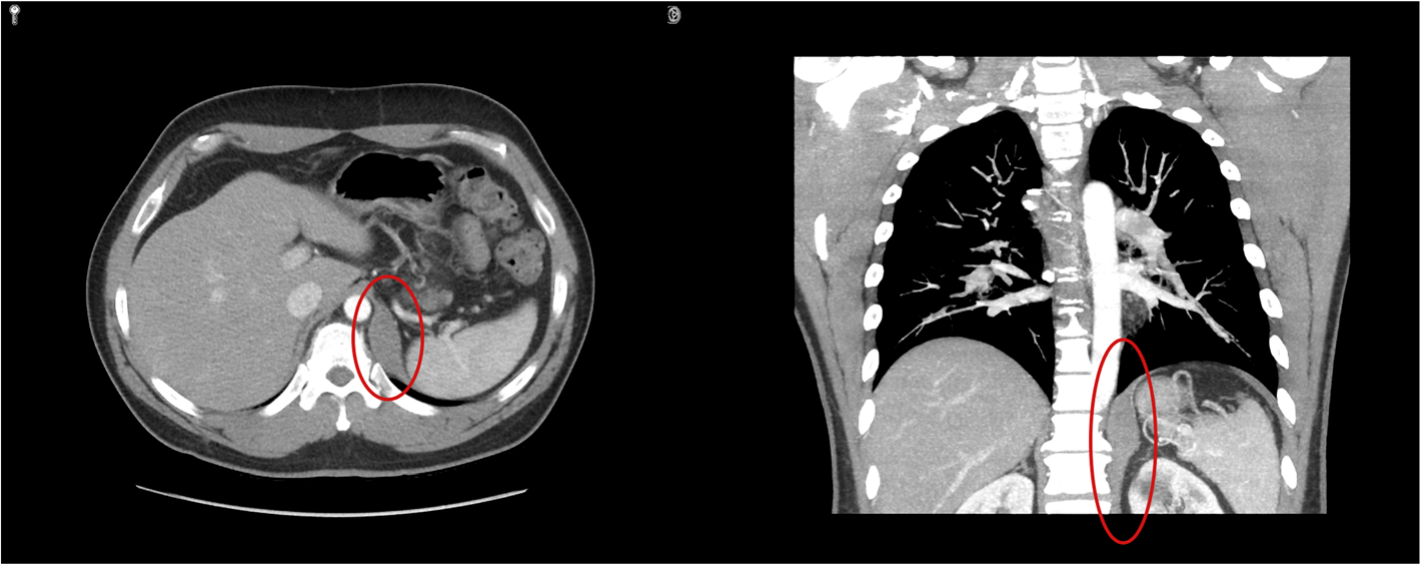
Axial and coronal slices of CT chest with contrast depicting a smoothly marginated and homogenous 5.3 cm × 2.3 cm × 5.9 cm soft tissue mass embedded within the muscle fibers of the crus of the left posterior medial hemidiaphragm with hounsfield of 60 units

A 41 year old male presented with 6 months of back pain located around his lumbar spine. He had no history of trauma. An MRI of the spine revealed an ovoid soft tissue structure located medially at the left lung base, measuring approximately 4.5 cm by 5 cm (Fig. 1). Due to its hyper intense signal, it was thought to possibly represent a bronchogenic or proteinaceous cyst. The mass was further characterized by a CT scan of the chest which demonstrated a homogeneous soft tissue mass embedded within the posterior medial crus of the left hemidiaphragm, measuring 5.3 × 2.3 × 5.9 cm (Fig. 2). An old CT had previously identified the mass and it had increased in size compared with ten years ago. The patient was otherwise asymptomatic with no complaints of chest pain, shortness of breath, weight loss, or abdominal pain. Physical examination of the patient revealed only mild mid thoracic spine tenderness.

The patient was taken to the operating room for a left posterolateral serratus sparing thoracotomy with resection of the left diaphragmatic mass. The diaphragm was reconstructed with a synthetic patch. The post-operative course was uneventful and the patient was discharged home on post-operative day five. Final pathology was consistent with a benign intradiaphragmatic bronchogenic cyst with clear margins. The mass was composed of benign ciliated respiratory epithelium, cartilage and seromucinous glands surrounded by benign skeletal muscle.

## Methods

We performed a combined MEDLINE, PubMed, ScienceDirect, and SCOPUS Database search for “bronchogenic cyst” AND “diaphragm” without date limitations. Case reports of intradiaphragmatic bronchogenic cysts were selected for analysis and review. Non-English language articles were excluded. Citations of these case reports were inspected for references to other case reports, and these were investigated for appropriateness and included if relevant. Patient demographics, case specifics, including imaging, and details of operative approach were noted in each case report and pooled together for analysis. If a particular case report did not include pertinent data, such as operative approach or diaphragmatic management, they were excluded from that particular analysis.

## Results

A total of twenty English language case reports for intradiaphragmatic bronchogenic cysts were published from 1952 to 2014. The age of presentation varied significantly and ranged from 19 months to 74 years old, with a mean age of 42.7 years old. 57 % (12/21) of the patients were female. 71.4 % (15/21) presented on the left side. Most frequently, presentation was either for back pain or cough, each of which were present in 19 % (4/21) (Table 1). Other common symptoms on presentation included chest pain, abdominal pain, or weight loss in 14 % (3/21) each. Prior to 1995, in the era pre-dating widespread tomography (CT), chest radiography (CXR) was the predominate mode of diagnosis. In the CT era, all cases involved CT at some point in the diagnostic workup, either when CXR was negative or to further characterize lesions seen on other modalities. CXR was negative in two patients with subsequent CT identifying the diaphragmatic lesion. Upper gastrointestial barium series and ultrasonography were also performed as part of patients’ workup, but were rarely diagnostic. The most likely preoperative diagnoses varied significantly, but these lesions were most commonly confused with adrenal masses (Table 2).

**Table 1:** Clinical Presentation

Presenting signs and symptoms	Percentage
Back pain	19 %
Cough	19 %
Asymptomatic	14 %
Chest pain	14 %
Abdominal pain	14 %
Weight Loss	14 %
Fatigue	10 %
Hiccups	10 %
Flank pain	10 %
Urgency	5 %
Incontinence	5 %
Nausea	5 %
Vomiting	5 %
Dyspnea	5 %
Fever of Unknown Origin	5 %

**Table 2:** Presumed Diagnosis Prior to Resection

Author	Year	Diagnosis
Elemen [6]	2008	Hydatid Cyst
Zugel [7]	2008	Symptomatic cystic liver tumor
Chang [8]	2006	Metastatic focus of hepatocellular carcinoma
Chang [8]	2006	Lung mass
Wesphal [9]	2003	Diaphragmatic hernia
Liou [10]	2001	Posterior mediastinal tumor
Desrumaux [11]	2001	Posterior mediastinal tumor
Hoang [12]	1999	Adrenal mass
Rozenblit [13]	1998	Adrenal cyst
Subramaniam [14]	1996	Adrenal tumor
Dagenais [15]	1995	Neurofibroma
Buddington [16]	1957	Adrenal mass
Kesseler [17]	1955	Diaphramatic cyst

When commented on, CT reports most commonly describe these lesions as homogeneous soft tissue hypoenhancing masses. The mean CT Hounsfield units were 38.3 ± 13.5. Calcification was present in three patients, and described as combined linear and nodular. MRI was performed in four patients to further characterize the lesions. MRI noted the majority to be T1/T2 hyperintense, but classification of them varied as either solid, soft tissue, or cystic lesions. The largest lesion was 10 cm and the mean size was 6.19 ± 2.58 cm.

Surgical approach varied, but was most commonly performed via a posterolateral thoracotomy (47.4 %; 9/19) (Table 3). Laparotomy, thoracoscopy, and a thoracoabdominal approach were the next most common utilized approach at 15.8 % (3/19) each, respectively (Table 4). All reported cases resulted in complete removal of the bronchogenic cyst. The specifics of reconstruction of the diaphragm were variable, and frequently not reported. Reconstruction was accomplished via primary closure in 69.2 % (9/13). Three cases (23.1 %) reported the use of prosthetic material to bridge a large diaphragmatic defect.

**Table 3:** Approaches to Resection

Surgical approach	Percent
Thoracotomy	47.4 % (9/19)
Laparotomy	15.8 % (3/19)
Thoracoabdominal	15.8 % (3/19)
Thoracoscopy	15.8 % (3/19)
Laparoscopy	5.3 % (1/19)

**Table 4:** Case Reports and Details

Author	Year	Age	Sex	Surgical Approach	Diaphragmatic Management	Comments
Mubang	2015	41	Male	Thoracotomy	Gore-tex patch reconstruction	Serratus sparing thoracotomy
Herek [18]	2014	42	Male	N/A	N/A	
Subramanian [19]	2013	13	Male	Thoracoscopy	Primary closure	Interrupted sutures
Jiang [20]	2013	38	Female	Thoracotomy	Primary closure	Interrupted figure of eight sutures
Kim [21]	2011	56	Female	Thoracotomy	Gore-tex patch reconstruction	Conversion from thoracoscopic approach
Elemen [6]	2008	1.6	Female	Laparotomy	No diaphragmatic injury	
Zugel [7]	2008	43	Female	Laparoscopy	Primary closure	Interrupted 2–0 Ethibond
Chang [8]	2006	74	Female	Thoracoscopy	N/A	
Chang [8]	2006	54	Female	Thoracoscopy	N/A	
Anile [22]	2006	38	Female	Thoracotomy	N/A	
Westphal [9]	2003	32	Female	Thoracotomy	Prolene mesh reconstruction	Non-absorbable sutures
Liou [10]	2001	34	Male	Thoracotomy	Primary closure	
Desrumaux [11]	2001	50	Male	N/A	N/A	
Hoang [12]	1999	51	Male	Laparotomy	N/A	
Rozenblit [13]	1998	64	Female	Laparotomy	N/A	
Subramaniam [14]	1996	50	Male	Thoracoabdominal	Primary closure	11th rib resection performed; interrupted 2–0 vicryl sutures
Dagenais [15]	1995	51	Female	Thoracoabdominal	Primary closure	
Gourlay [23]	1966	41	Male	Thoracotomy	Primary closure	Interrupted silk sutures
Aaron [24]	1964	21	Female	Thoracotomy	Primary closure	Two layer closure
Buddington [16]	1957	62	Male	Thoracoabdominal	Primary closure	Silk mattress sutures
Kesseler [17]	1955	41	Female	Thoracotomy	N/A	

On gross examination, six (28.6 %) were noted to be multilocular, while three (14.3 %) were unilocular, with the remaining not describing loculations. Four cases (19.0 %) described calcification present in specimen, three noting the calcification to be focal. One of these cases did not show radiologic evidence of calcification. All pathologic analysis denoted absence of malignancy; however, two cases denoted the presence of squamous metaplasia within the resected bronchogenic cyst. Sample size would limit any analysis of predictors of metaplasia in this series.

## Discussion

Bronchogenic cysts are congenital lesions that are thought to arise from an abnormally budding ventral foregut, which then develops into a blind ending fluid filled pouch [1]. However, the exact embryologic development of these cysts is currently unclear [2]. Given that they likely arise from embryologic error, bronchogenic cysts are also associated with other congenital pulmonary malformations, such as congenital lobar emphysema or pulmonary sequestration [1]. On occasion, bronchogenic cysts may harbor malignancy, and the presence of a bronchogenic cyst necessitates surgical resection [3]. While commonly located in the mediastinum, the intradiaphragmatic location is exceedingly rare, and our case documents the twenty-first case in the English literature [1].

Symptoms that are seen are frequently nonspecific and may not be related to the bronchogenic cyst itself, but rather prompting workup, which then reveals the lesion. When symptoms arise, typically patients present with pain, but they can also have respiratory symptoms such as intermittent or persistent cough. Symptoms are likely due to compression or irritation of adjacent structures. Bronchogenic cysts, as a group, can also present with symptoms from cyst communication with aerodigestive structures, bleeding, or localized infections, but none of these were present with bronchogenic cysts in the intradiaphragmatic location [1]. Imaging studies have an increasingly important role in the diagnosis or suggestion of intradiaphragmatic bronchogenic cysts. Compared with mediastinal bronchogenic cysts, intradiaphragmatic lesions have similar CT and MRI characteristics, but they are not completely diagnostic. A combined approach utilizing multiple modalities of imaging may help to characterize these lesions, but given their rarity, are often difficult diagnoses to make on imaging alone. Due to this, surgical resection remains necessary for diagnosis [4].

It appears as though surgical approaches for resection of these lesions are dependent on the most likely diagnosis as well as the anticipated extent of diaphragmatic reconstruction. Frequently, preoperative localization of these lesions are difficult, and they may be mistakenly thought to be either within the abdominal or thoracic cavities, rather than within the diaphragm itself. However, our review notes that virtually all approaches are suitable for successful resection of intradiaphragmatic bronchogenic cysts, including minimally invasive approaches. We can speculate that minimally invasive approaches would yield improved short term outcomes, but with such a small sample size, and each unique case presenting its own challenges, we could only speculate as to that conclusion.

The pathologic hallmark of bronchogenic cysts is the presence of ciliated pseudostratified columnar epithelium, cartilage, and smooth muscle within the cyst wall. Grossly, there is a variable presentation, which likely contributes to their variable radiologic appearance. Reason for resection of these lesions is their unclear diagnosis, potential for harboring malignancy, infectious complications, erosive complications, and symptomatology, if present [1]. While pathologic analysis did not reveal evidence of malignancy present in any of the cases, the presence of squamous metaplasia in two cases, suggests their malignant potential and raises the possibility of stepwise carcinogenesis in these lesions [5].

## Conclusions

Intradiaphragmatic bronchogenic cysts are very rare, and this review characterizes the variable nature of clinical presentation, radiologic appearance, surgical techniques, and pathology. These lesions should remain on the differential diagnosis in cases of unusual masses in the region of the diaphragm.

## References

[1] Sugarbaker David J (2009). "Overview of Benign Lung Disease: Anatomy and Pathophysiology." *Adult Chest Surgery*.

[2] Liou C-H, Hsu H-H, Hsueh C-J, Juan C-J, Chen C-Y (2001). Imaging Findings of Intradiaphragmatic Bronchogenic Cyst: A Case Report. Journal of Formosan Medical Association.

[3] Endo C, Imai T, Nakagawa H, Ebina A, Kaimori M (2000). Bronchioloalveolar Carcinoma Arising in a Bronchogenic Cyst. The Annals of Thoracic Surgery.

[4] Page Mcadams H, Kirejczyk WM, Rosado-De- Christenson ML, Shigeru M (2000). Bronchogenic Cyst: Imaging Features with Clinical and Histopathologic Correlation1. Radiology.

[5] Ooi AT, Gower AC, Zhang KX, Vick JL, Hong L, Nagao B, et al. Molecular Profiling of Premalignant Lesions in Lung Squamous Cell Carcinomas Identifies Mechanisms Involved in Stepwise Carcinogenesis. Cancer Prevention Research. 2014;7(5):487–95. Web. 10.1158/1940-6207.CAPR-13-0372 PMC4059064 24618292

[6] Elemen L, Tugay M, Tugay S, Gürcan NI, Erkus B, Gurbuz Y (2008). Bronchogenic Cyst of the Right Hemidiaphragm Mimicking a Hydatid Cyst of the Liver: Report of the First Pediatric Case. Pediatr Surg Int Pediatric Surgery International.

[7] Zugel N, Kox M, Lang R, Huttl T (2008). Laparoscopic Resection of an Intradiaphragmatic Bronchogenic Cyst. Journal of Society of Laparoendoscopic Surgeons.

[8] Chang Y-C, Jin-Seng C, Yih-Leong C, Yung-Chie L (2006). Video-Assisted Thoracoscopic Excision of Intradiaphragmatic Bronchogenic Cysts: Two Cases. Journal of Laparoendoscopic & Advanced Surgical Techniques.

[9] Westphal FL, Menezes AQ, Gonçalves Guimarães RA. Intradiaphragmatic Bronchogenic Cyst. J. Pneumologia Jornal De Pneumologia. 29.3 (2003): n. pag. Web. http://dx.doi.org/10.1590/S0102-35862003000300007.

[10] Liou C-H, Hsu H-H, Hsueh C-J, Juan C-J, Chen C-Y. Imaging Findings of Intradiaphragmatic Bronchogenic Cyst: A Case Report. Journal of Formosan Medical Association. 2001;100(10):712–14. Web. 11760380

[11] Desrumaux I, De Weaver W, Verschakelen J (2001). Paravertebral and Diaphragmatic Mass: An Ectopic Location of Bronchogenic Cyst. Journal of Belgian Society of Radiology.

[12] Hoang C (1999). Retroperitoneal Intradiaphragmatic Bronchogenic Cyst.". Journal of Clinical and Experimental Pathology..

[13] Rozenblit A (1998). AIqbal, R Kaleya, G Rozenblit. Intradiaphragmatic Bronchogenic Cyst. Clinical Radiology..

[14] Subramaniam P (1996). A Scott. A Case of Congenital Bronchogenic Cyst in a Rare Intradiaphragmatic Position Mimicking a Left Adrenal Tumor. The Asia Pacific Heart Journal.

[15] Dagenais F (1995). E Nassif, R Déry, R Lapointe. Bronchogenic Cyst of the Right Hemidiaphragm. The Annals of Thoracic Surgery.

[16] Buddington WT (1957). Intradiaphragmatic Cyst. New England Journal of Medicine N Engl J Med..

[17] Kesseler HJ, Maier HC (1955). Intradiaphragmatic Cysts. Journal of Thoracic Surgery..

[18] Herek D, Erbis H, Kocyigit A, Baki Yagci A. Retroperitoneal Bronchogenic Cyst Originating from Diaphragmatic Crura. Indian Journal of Surgery*.* 2014; n. pag. Web. 10.1007/s12262-014-1045-2 PMC4775676 27011575

[19] Subramanian S, Tushar C, Whitehouse J, Suchi M, Arca M, Maheshwari M (2013). Bronchogenic Cyst in the Intradiaphragmatic Location. WMJ.

[20] Jiang C, Wang H, Chen G, Jiang G, Zhang P (2013). Intradiaphragmatic Bronchogenic Cyst. The Annals of Thoracic Surgery.

[21] Kim JB, Park C-K, Kum D-Y, Lee D-H, Jung HR (2011). Bronchgenic Cyst of the Right Hemidiaphragm Presenting with Pleural Effusion. Korean Journal of Thoracic and Cardiovascular Surgery.

[22] Anile M, Di Stasio M, Vitolo D, Venuta F (2006). Intradiaphragmatic Bronchogenic Cyst. European Journal of Cardio-Thoracic Surgery.

[23] Gourlay RH, Aspinall RJ. Bronchogenic Cyst of the Diaphragm: A Case Report. Canadian Journal of Surgery. 1966;169–72. Web. 5937197

[24] Aaron BL (1969). Intradiaphragmatic Cyst: A Rare Entity. Journal of Thoracic and Cardiovascular Surgery.

